# Integrated mRNA-seq and miRNA-seq analysis reveals miR-210a-5p regulates uterine aging in laying hens by targeting the RASL11B/Raf/MAPK pathway

**DOI:** 10.1186/s40104-025-01257-y

**Published:** 2025-09-23

**Authors:** Xiyu Zhao, Xinyan Li, Wenxin Zhang, Mingyue Gao, Conghao Zhong, Boxuan Zhang, Congjiao Sun, Yao Zhang, Shunshun Han, Huadong Yin

**Affiliations:** 1 State Key Laboratory of Swine and Poultry Breeding Industry, Key Laboratory of Agricultural Bioinformatics, Ministry of Education, Key Laboratory of Livestock and Poultry Multi-Omics, Ministry of Agriculture and Rural Affairs, and, Farm Animal Genetic Resources Exploration and Innovation Key Laboratory of Sichuan Province, College of Animal Science and Technology , Sichuan Agricultural University, 611130 Sichuan, People’s Republic of China; 2 State Key Laboratory of Animal Biotech Breeding, Frontier Science Center of Molecular Design Breeding, China Agricultural University, Beijing, 100193 China

**Keywords:** Epithelial senescence, Laying hen, MAPK pathway, MiR-210a-5p, RASL11B, Uterine aging

## Abstract

**Background:**

Uterine aging is a key factor contributing to the deterioration of egg quality and reproductive performance in laying hens. Despite its importance, the molecular mechanisms underlying uterine aging remain poorly defined. This study aimed to characterize gene expression and regulatory changes associated with uterine aging in hens at different life stages.

**Results:**

Transcriptomic Analysis of uterine tissue from hens aged 350, 500, And 700 d revealed dynamic changes in gene expression patterns during aging. A significant upregulation of genes involved in cellular senescence was observed, including increased expression of the p53 signaling pathway And markers associated with inflammation And cell cycle arrest. The most notable changes occurred between 350 And 500 d of age, suggesting this as a critical window for the onset of uterine aging. MicroRNA sequencing identified miR-210a-5p as significantly reduced with age. Target prediction and experimental validation showed that miR-210a-5p directly suppresses the expression of RASL11B, a Ras-like small GTPase that activates the MAPK signaling pathway. In primary uterine epithelial cells, reduced miR-210a-5p levels led to elevated RASL11B expression, increased activation of B-Raf, MEK, and ERK proteins, and enhanced expression of aging-related genes and inflammatory factors. In contrast, overexpression of miR-210a-5p or inhibition of the MAPK pathway delayed senescence and reduced inflammatory signaling. RASL11B overexpression was sufficient to induce aging phenotypes, confirming its central role in promoting uterine cellular aging.

**Conclusions:**

This study identifies a novel regulatory pathway in which miR-210a-5p modulates uterine aging through the RASL11B-MAPK signaling cascade. The findings provide mechanistic insight into age-related reproductive decline in hens and suggest that targeting this pathway may offer new strategies for maintaining uterine function and extending reproductive lifespan in poultry.

**Supplementary Information:**

The online version contains supplementary material available at 10.1186/s40104-025-01257-y.

## Background

Eggshell quality plays a vital role in the efficiency of egg production in poultry, directly affecting both the commercial value and the welfare of laying hens [1]. A key determinant of eggshell quality is the health of the oviduct, specifically the uterine section, where calcium carbonate is deposited to form the eggshell [2, 3]. As hens age, a progressive decline in the function of the uterus leads to reduced eggshell quality, manifesting as thinner and weaker shells. This decline not only compromises the integrity of eggs but also hampers the overall productivity of the flock, presenting significant economic challenges to poultry farming [4]. The uterine aging process is influenced by a variety of factors, including cellular senescence, oxidative stress, and hormonal changes, which impair the tissue’s ability to secrete the essential proteins and minerals required for shell formation [5]. Given the importance of the uterus in this process, understanding the molecular mechanisms underlying uterine aging and its impact on eggshell quality is crucial for improving egg production efficiency and prolonging reproductive lifespan in laying hens.

Recent advances in high-throughput sequencing technologies have provided powerful tools for exploring the complex molecular landscape of aging in reproductive tissues. These technologies enable the identification of genes and regulatory pathways that may contribute to the aging process at a transcriptomic and post-transcriptional level [6]. In this context, microRNAs (miRNAs), small non-coding RNA molecules, have emerged as key regulators of gene expression, modulating a wide array of biological processes including cell proliferation, apoptosis, and aging [7–9]. Previous studies have shown that miR-29 is markedly upregulated during the aging process in mice and plays a pivotal role in regulating gene expression programs that drive age-associated phenotypes [10, 11]. Moreover, miR-708 has been identified as an aging-suppressive miRNA, with decreased expression in senescent cells and aged tissues [12].

One of the primary mechanisms by which miRNAs exert their regulatory effects is through the competing endogenous RNA (ceRNA) network. In this model, miRNAs bind to the 3′ untranslated regions (3′ UTRs) of target mRNAs, thereby repressing their translation or promoting their degradation [13]. This interaction not only alters the expression levels of specific target genes but also affects the balance of ceRNA molecules that compete for the same miRNA pool [14, 15]. As a result, changes in miRNA activity can have widespread effects on gene expression networks. Importantly, the downstream impact of miRNA-mediated gene regulation often extends to multiple signaling pathways, as many target genes act as key nodes or regulators within these pathways. Alterations in the expression of such target genes can initiate or suppress signaling cascades, thereby influencing cellular senescence [16]. For instance, miR-34a induces cellular senescence in mice by targeting SIRT1, a longevity-associated deacetylase involved in stress resistance, and downregulates antioxidant pathways, thereby amplifying oxidative stress-induced damage [17]. Additionally, miR-146a, studied primarily in human fibroblasts, inhibits the secretion of senescence-associated secretory phenotype (SASP) components, mitigating chronic inflammation and delaying senescence [18]. Another study found that, in addition to the classical p53/p21 pathway, the p53/miRNAs/CCNA2 axis functions as an important regulatory mechanism in cellular senescence. In this pathway, aging-associated miRNAs such as miR-124, miR-34a, and miR-29a/b/c, which have been validated in mouse embryo fibroblasts, are transcriptionally upregulated by p53 and target Ccna2, a key cell cycle promoter. The downregulation of Ccna2 reinforces p53-driven cell cycle arrest, offering an additional layer of senescence control [19]. Thus, miRNAs, through the ceRNA mechanism, represent a critical layer of regulatory control over complex signaling networks, including those involved in the aging process [20].

In this study, we employed an integrated mRNA sequencing (mRNA-seq) and miRNA sequencing (miRNA-seq) approach to delineate the miRNA-mRNA regulatory network underlying uterine aging in laying hens. Our analysis identified miR-210a-5p as a critical regulator that targets *RASL11B*, thereby modulating the Raf/MAPK signaling pathway. Collectively, these findings provide new insights into the molecular regulation of uterine aging in laying hens and highlight the miR-210a-5p/RASL11B/Raf/MAPK axis as a promising target for interventions aimed at improving reproductive longevity and production efficiency.

## Materials and methods

### Animal ethics and sample collection

A total of 120 Jingfen No.6 laying hens were used in this study. All birds were obtained from Beijing Huadu Yukou Poultry Breeding Co., Ltd. (China). The hens were individually housed in standard laying cages under controlled environmental conditions with ad Libitum access to a commercial layer diet And clean water. The Lighting regime was maintained at 16 h of Light And 8 h of darkness per day (16L:8D). Birds were randomly selected And euthanized at three time points corresponding to different stages of the laying cycle: 350 d (early senescence stage), 500 d (mid senescence stage), And 700 d (late senescence stage). At each time point, six birds were used for tissue collection and subsequent experiments. After euthanasia, the oviduct was rapidly dissected and the uterine segment (shell gland) was isolated. The tissues were Weighed And measured for gross morphological assessment. Samples were either fixed in 4% paraformaldehyde for histological and immunohistochemical analysis, or immediately snap-frozen in liquid nitrogen and stored at −80 °C for RNA, protein extraction, and sequencing. All experimental procedures involving animals in this study were conducted in strict accordance with the Animal Management Regulations of the Welfare Committee of Sichuan Agricultural University (Approval number: 2023102023).

### Egg quality measurements

At 350, 500, And 700 days of age, 20 eggs were randomly collected to evaluate egg quality parameters, including eggshell thickness and eggshell strength. Eggshell thickness was measured using a micrometer at three locations (blunt end, equator, and sharp end) and averaged. Eggshell strength was assessed using an eggshell force gauge (Bulader, Beijing, China), and data were recorded in Newtons.

### Histology and immunohistochemistry (IHC)

Uterine tissues were fixed in 4% paraformaldehyde, dehydrated, embedded in paraffin, And sectioned at a thickness of 5 μm. For histological evaluation, sections were stained with hematoxylin and eosin (H&E) to assess structural changes in the uterine epithelium. For immunohistochemical analysis, paraffin sections were deparaffinized, rehydrated, and subjected to antigen retrieval. Sections were then incubated with primary antibodies against Lamin B and p53 (ABclonal, Wuhan, China), followed by incubation with horseradish peroxidase-conjugated secondary antibodies (ABclonal). The detailed information of all antibodies used was shown in Table S1. Signal detection was performed using diaminobenzidine as the chromogen. Nuclei were counterstained with hematoxylin. Stained sections were visualized and imaged under a light microscope (Olympus, Tokyo, Japan).

### RNA isolation and quantitative real-time PCR (qPCR)

Total RNA from uterine tissues and cultured cells was extracted using TRIzol reagent (Takara, Otsu, Japan) according to the manufacturer’s protocol. cDNA synthesis was performed using PrimeScript RT reagent kit (Takara). qPCR was carried out with SYBR Green Master Mix (Takara) on a CFX Connect™ Real-Time PCR Detection System (Bio-Rad, Hercules, USA). Expression levels were normalized to GAPDH And Analyzed using the 2^–ΔΔCt^ method. Primer sequences were provided in Table S2.

### mRNA and miRNA sequencing

For transcriptomic analysis, RNA from uterine tissues of three laying hens per age group was used. For mRNA sequencing, poly(A)^+^ RNA was enriched using oligo(dT) magnetic beads, followed by fragmentation, first and second-strand cDNA synthesis, end repair, A-tailing, adapter Ligation, And PCR amplification to construct sequencing Libraries. For miRNA sequencing, small RNAs of 18–30 nt were isolated, Ligated to 5′ And 3′ adapters, reverse transcribed, and amplified to generate cDNA Libraries. Library quality was verified using the Agilent 2100 system. All Libraries were sequenced on the Illumina NovaSeq 6000 platform to generate 150 bp paired-end reads (for mRNA-seq) and single-end reads (for miRNA-seq). Sequencing was performed by Novogene (Beijing, China). Raw sequencing data were subjected to quality control, adapter trimming, and filtering of low-quality reads. Clean reads were aligned to the chicken reference genome (GRCg7b) using HISAT2 for mRNA-seq and Bowtie for miRNA-seq. Expression levels were quantified as fragments per kilobase of transcript per million mapped reads (FPKM) for mRNA and transcripts per million (TPM) for miRNA. Differentially expressed genes (DEGs) and miRNAs (DEMs) were identified using DESeq2 with thresholds of |log₂FC| ≥ 1 and *Q*-value < 0.01. To assess the robustness of differential expression, we calculated statistical power and false positive rate (FPR) using the PROPER R package [21]. Heatmap analysis was based on the FPKM values of genes and was performed with row normalization. Principal component analysis (PCA) was used to assess overall differences among age groups. Target genes of miRNAs were predicted using miRDB and RNAhybird. Volcano plots and heatmaps were generated in R to visualize expression patterns. Gene Ontology (GO) and Kyoto Encyclopedia of Genes and Genomes (KEGG) pathway enrichment analyses were conducted using clusterProfiler, with adjusted *P*-values < 0.05 considered statistically enriched.

### Dual-luciferase reporter assay

The 3′ UTR of *RASL11B* containing the predicted miR-210a-5p binding site was cloned into the pmirGLO vector (RASL11B-WT). Mutations were introduced using site-directed mutagenesis (RASL11B-MT). DF-1 cells were co-transfected with miR-210a-5p mimic or negative control (NC) And reporter constructs. Luciferase activity was measured 48 h post-transfection using the Dual-Luciferase Reporter Assay System (Beyotime, Shanghai, China).

### Primary uterine epithelial cell (UEC) culture

Fresh uterine tissues were collected from healthy laying hens and rinsed with sterile phosphate buffer saline (PBS). After removing connective And unrelated tissues, the uterus was opened, And the endometrium was gently peeled and minced. Tissue fragments were digested with 1 mg/mL type I collagenase at 37 °C for 60 min with intermittent shaking. Digestion was terminated with complete F12 medium (F12 + 10% Fetal bovine serum + 1% penicillin–streptomycin). The cell suspension was filtered through a 70-μm strainer And centrifuged at 400 × *g* for 5 min. Cells were resuspended in F12 medium And seeded for differential adhesion. After 3 h, non-adherent cells were transferred to fresh plates. Medium was changed after 48 h to remove residual non-epithelial cells. Cell identity was confirmed by PCR for epithelial marker genes and immunofluorescence (IF) staining for ESRα and KRT18, which showed over 95% positivity, confirming high purity of isolated UECs.

### IF staining

Isolated UECs were seeded on poly-L-lysine-coated coverslips, fixed with 4% paraformaldehyde, And permeabilized using 0.2% Triton X-100. After blocking with 5% BSA, cells were incubated overnight at 4 °C with primary antibodies against ESRα and KRT18 (ABclonal, Table S3). After washing, fluorescent secondary Antibodies were applied for 1 h at room temperature. Nuclei were counterstained with DAPI. Images were acquired using a fluorescence microscope (Olympus).

### Synthesis of RNA oligonucleotides and vector construction

Synthetic miR-210a-5p mimics, inhibitors, and corresponding NCs were designed and synthesized by GenePharma (Shanghai, China). Small interfering RNAs (siRNAs) targeting *RASL11B* were also synthesized by the same company. The sequences of all oligonucleotides were provided in Table S3. For overexpression studies, the full-length coding sequence of *RASL11B* was amplified and cloned into the pcDNA3.1(+) vector (Geneseed, Guangzhou, China). All constructs were verified by sequencing. Transfections were performed using Lipofectamine 3,000 (Invitrogen, Waltham, USA) according to the manufacturer’s instructions. Transfection efficiency was confirmed by qPCR 24 h post-transfection.

### Western blot assay

Proteins were extracted using RIPA buffer (BestBio, Shanghai, China), quantified, and separated on SDS-PAGE. After transfer to polyvinylidene fluoride membranes (Beyotime, Shanghai, China), blots were incubated with primary antibodies against p53, p21, MDM2, B-Raf, phospho-B-Raf, MEK, phospho-MEK, ERK, and phospho-ERK (ABclonal), followed by HRP-conjugated secondary antibodies (ABclonal). The detailed information of all antibodies used was shown in Table S1. Detection was performed using enhanced chemiluminescence (Beyotime). Images were acquired using the ChemiDoc XRS + imaging system (Bio-Rad), and band intensities were quantified using ImageJ software.

### SA-β-galactosidase (β-gal) staining

For SA-β-gal staining, a senescence detection kit (Beyotime) was used according to manufacturer’s instructions. Cells were first rinsed thoroughly with PBS And fixed in 4% paraformaldehyde at room temperature for 15 min, followed by three washes with PBS. A staining solution containing X-Gal was then added (1 mL/well in a 12-well plate), And the cells were incubated 60 min at 37 °C. After incubation, cells were washed with PBS to remove excess stain and minimize non-specific background. Images were captured from three random fields per group using an inverted microscope, and the average ratio of β-gal-positive area was calculated using ImageJ software.

### Enzyme linked immunosorbent assay (ELISA)

Interleukin-1β (IL-1β), IL-6, IL-8, and tumor necrosis factor α (TNF-α) levels in cell supernatants were quantified using ELISA kits (R&D, Minneapolis, USA), following the manufacturer’s instructions. Absorbance was measured at 450 nm using a microplate reader (Thermo Fisher, Carlsbad, USA).

### Data statistics and analysis

All experiments were performed in triplicate unless otherwise stated. Data are presented as the mean ± standard error of the mean (SEM). Statistical Analyses were conducted using SPSS 19 software. Differences between two groups were assessed using an unpaired Student’s *t*-test, while comparisons among multiple groups were analyzed using one-way ANOVA followed by Tukey’s multiple comparison test. A *P*-value < 0.05 was considered statistically significant, while *P*-value < 0.01 was considered highly significant.

## Results

### Age-related degeneration of the oviduct in layers during the late laying period

**Fig. 1:**
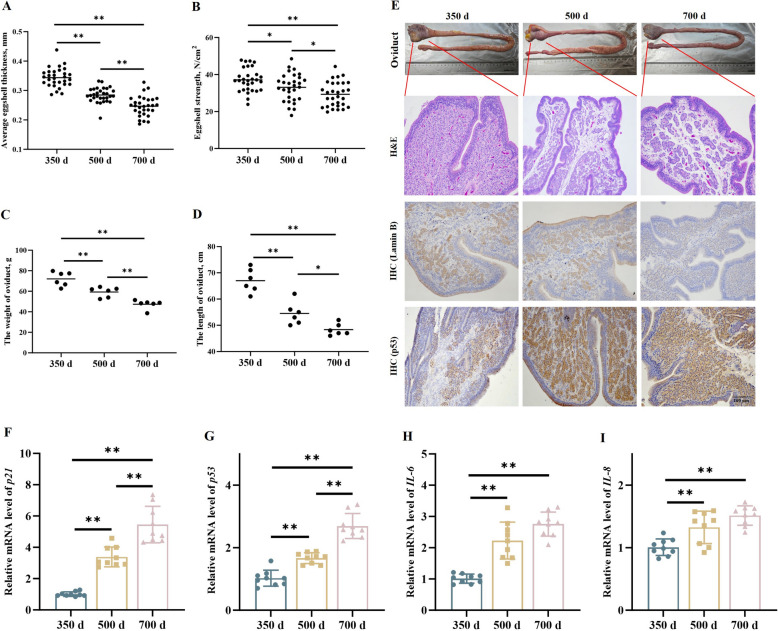
Morphological and molecular characterization of uterine aging in laying hens. **A** and** B** Eggshell thickness and strength were significantly reduced in older hens. **C** and** D** The weight and length of the oviduct decreased significantly with age. **E** H&E staining of uterine tissues at 350, 500, And 700 d showing progressive glandular atrophy and epithelial disorganization. Immunohistochemical staining of Lamin B and p53 in uterine tissues, indicating age-related decrease in Lamin B and increase in p53 expression. **F**–**I** qPCR results showing increased mRNA levels of *p21*, *p53*, *IL-6*, and *IL-8* with age . *P < 0.05, **P < 0.01

Firstly, egg quality assessment revealed that both eggshell thickness and eggshell strength significantly declined with increasing age during the late laying period (*P* < 0.01, Fig. 1A and B). To investigate age-associated changes in the oviduct, we subsequently measured the Weight And length of oviducts from laying hens at 350, 500, And 700 d of age. The results indicated a progressive decline in both parameters with advancing age (*P* < 0.01, Fig. 1C and D). Histological analysis using H&E staining revealed glandular atrophy, disorganized epithelial cell arrangement, and Functional deterioration in the uterine segment at 500 And 700 d (Fig. 1E). IHC staining showed a decrease in Lamin B expression and an increase in p53 expression with age (*P* < 0.01, Fig. 1E, Fig. S1). Moreover, qPCR analysis demonstrated elevated mRNA levels of senescence markers *p21* and *p53*, as well as pro-inflammatory cytokines *IL-6* and *IL-8*, correlating with age (*P* < 0.01, Fig. 1F–I). Collectively, these findings suggest that the uterine segment of the oviduct exhibits senescent phenotypes during the late laying period.​

### mRNA sequencing analysis of the uterine segment in aged laying hens

**Fig. 2:**
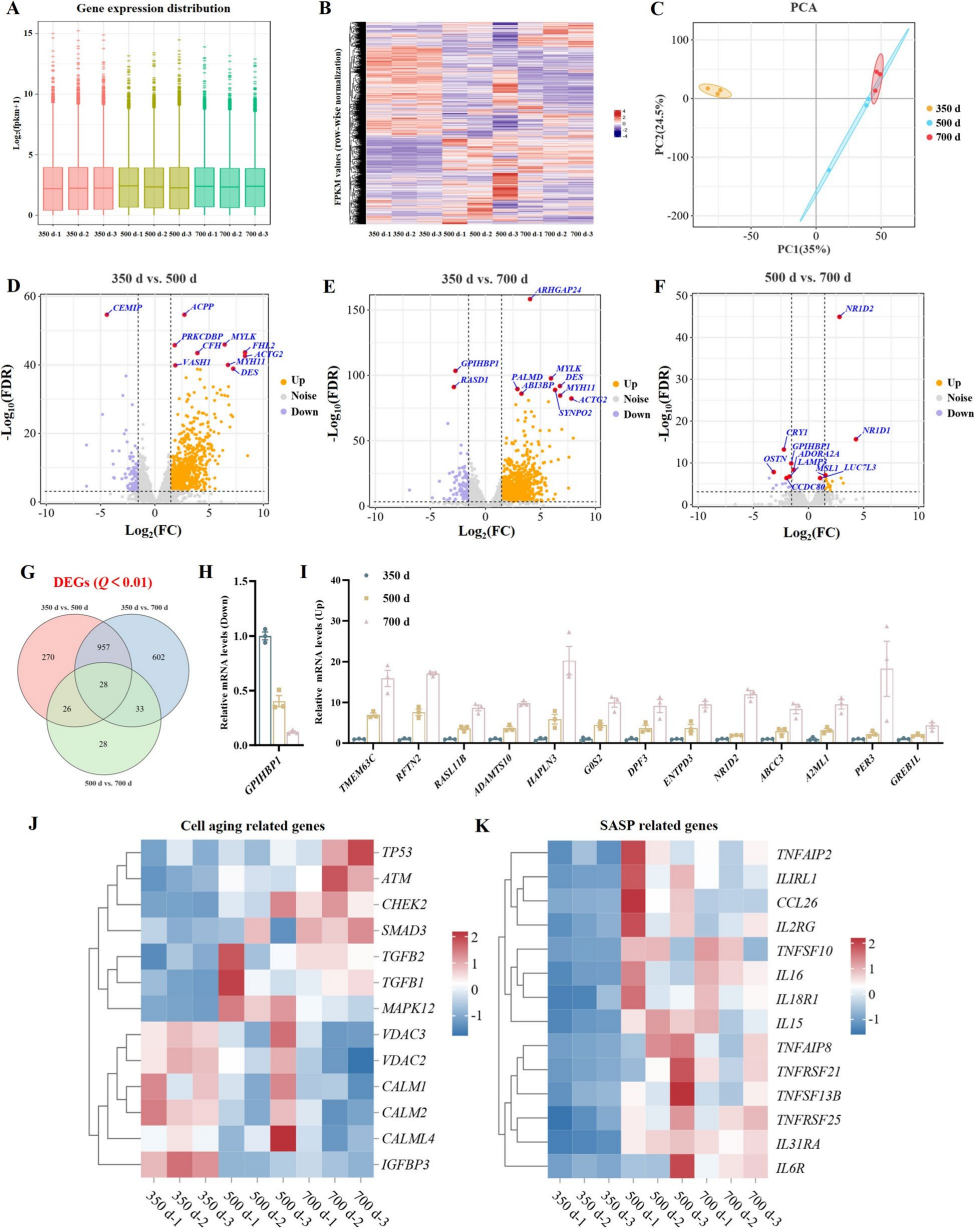
Transcriptomic alterations in aged uterine tissues. **A** Gene expression distribution across hens aged 350, 500, And 700 d.** B** Heatmap of representative upregulated and downregulated genes during aging. **D**–**F** Volcano map of DEGs among 350, 500 And 700 d. **G** Venn diagram identifying overlapping DEGs across all comparisons. **H** and** I** The relative expression levels of genes commonly differentially expressed across the three time points. **J** and **K** Heatmap of senescence-associated and SASP genes

To elucidate transcriptional changes associated with oviduct aging, we performed mRNA-seq on uterine tissues from hens aged 350, 500, And 700 d. PROPER analysis indicated that the estimated FPR across all comparisons remained consistently low (~ 0.0078), suggesting effective control of type I error. Gene expression distribution plots indicated consistent overall expression across samples, with a slight decline in transcriptional activity observed with age (Fig. 2A). Heatmap analysis identified significant temporal changes in gene expression patterns, implicating pathways related to senescence, uterine degeneration, and immune regulation (Fig. 2B). PCA revealed distinct separation between 350 d And 500/700 d, suggesting systemic transcriptional reprogramming during aging (Fig. 2C). Volcano plots showed a greater number of upregulated genes compared to downregulated ones with age, particularly between 350 And 500 d, indicating this interval as a critical period for uterine aging (Fig. 2D–F). Venn diagram Analysis identified 28 genes differentially expressed across all three time points, including 1 downregulated And 13 upregulated with age (Fig. 2G–I). Heatmap analysis of senescence-associated genes across different age groups revealed an age-dependent increase in the expression of key regulators such as *TP53* and *ATM*. In contrast, genes involved in cell cycle progression, mitochondrial function, and cellular homeostasis exhibited progressively decreased expression with advancing age (Fig. 2J). Genes associated with the SASP were significantly upregulated at 500 And 700 d (Fig. 2K).

**Fig. 3:**
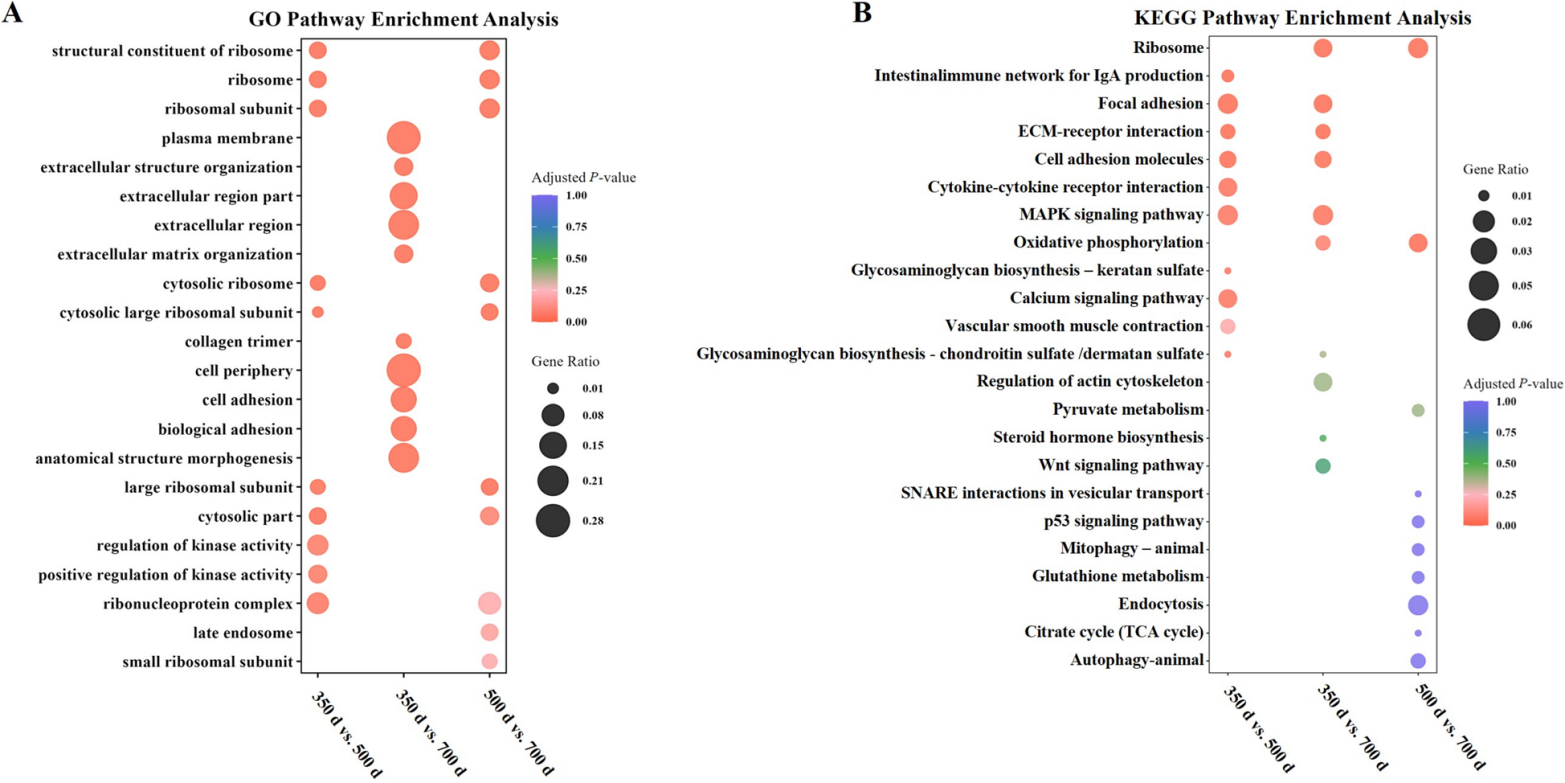
Enrichment analysis of DEGs during the process of uterine aging. **A** GO enrichment analysis of DEGs during uterine aging. **B** KEGG pathway enrichment analysis of DEGs during uterine aging

GO enrichment analysis highlighted age-associated changes in ribosome-related processes, extracellular matrix organization, cell adhesion, and enzyme activity modulation (Fig. 3A). KEGG pathway analysis revealed significant enrichment in several key pathways related to cellular function and signaling. These included the ribosome pathway, oxidative phosphorylation, and cell adhesion. Additionally, pathways involved in immune response such as the intestinal immune network for IgA production and cytokine-cytokine receptor interaction were enriched. Other notable enriched pathways included ECM-receptor interaction, MAPK signaling, and calcium signaling pathways, which are critical for cellular communication and regulation (Fig. 3B). These results suggest that the period between 350 And 500 d is critical for uterine aging in laying hens, characterized by significant transcriptional alterations and senescence features.​

### miRNA sequencing analysis of the uterine segment during aging

**Fig. 4:**
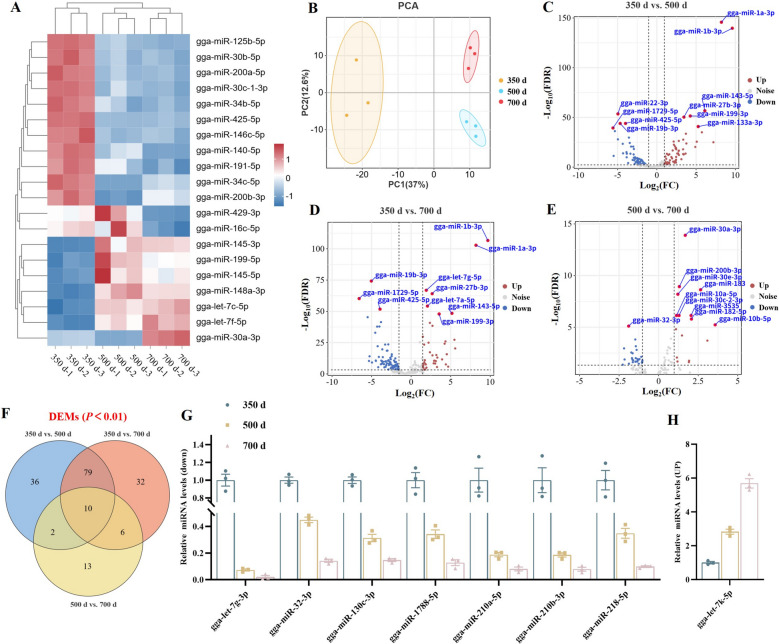
Age-dependent changes in miRNA expression in uterine tissues. **A** Heatmap of top 20 DEMs across age groups. **B** PCA showing distinct clustering by age. **C**–**E** Volcano map of DEGs among 350, 500 And 700 d. **F** Venn diagram identifying overlapping DEMs across all comparisons. **G** and** H** The expression levels of miRNAs commonly differentially expressed across the three time points

To explore the miRNA regulatory network involved in uterine aging, we conducted miRNA-seq on uterine tissues from hens aged 350, 500, And 700 d. The top 20 differentially expressed miRNAs were identified (Fig. 4A). PCA demonstrated clear separation among the three age groups, with the most significant differences between 350 And 500 d (Fig. 4B). Volcano plot Analysis revealed more downregulated miRNAs than upregulated ones with age, particularly between 350 And 500 d, reinforcing this interval as a key period for uterine aging (Fig. 4C–E). Venn diagram Analysis identified 10 miRNAs differentially expressed across all three time points, with 7 downregulated And 1 upregulated with age (Fig. 4F–P). Furthermore, 79 miRNAs were found to be commonly differentially expressed in both the 350 d vs. 500 d And 350 d vs. 700 d comparisons. These shared miRNAs are likely to play crucial roles in the early phases of uterine aging (Fig. 4F).

**Fig. 5:**
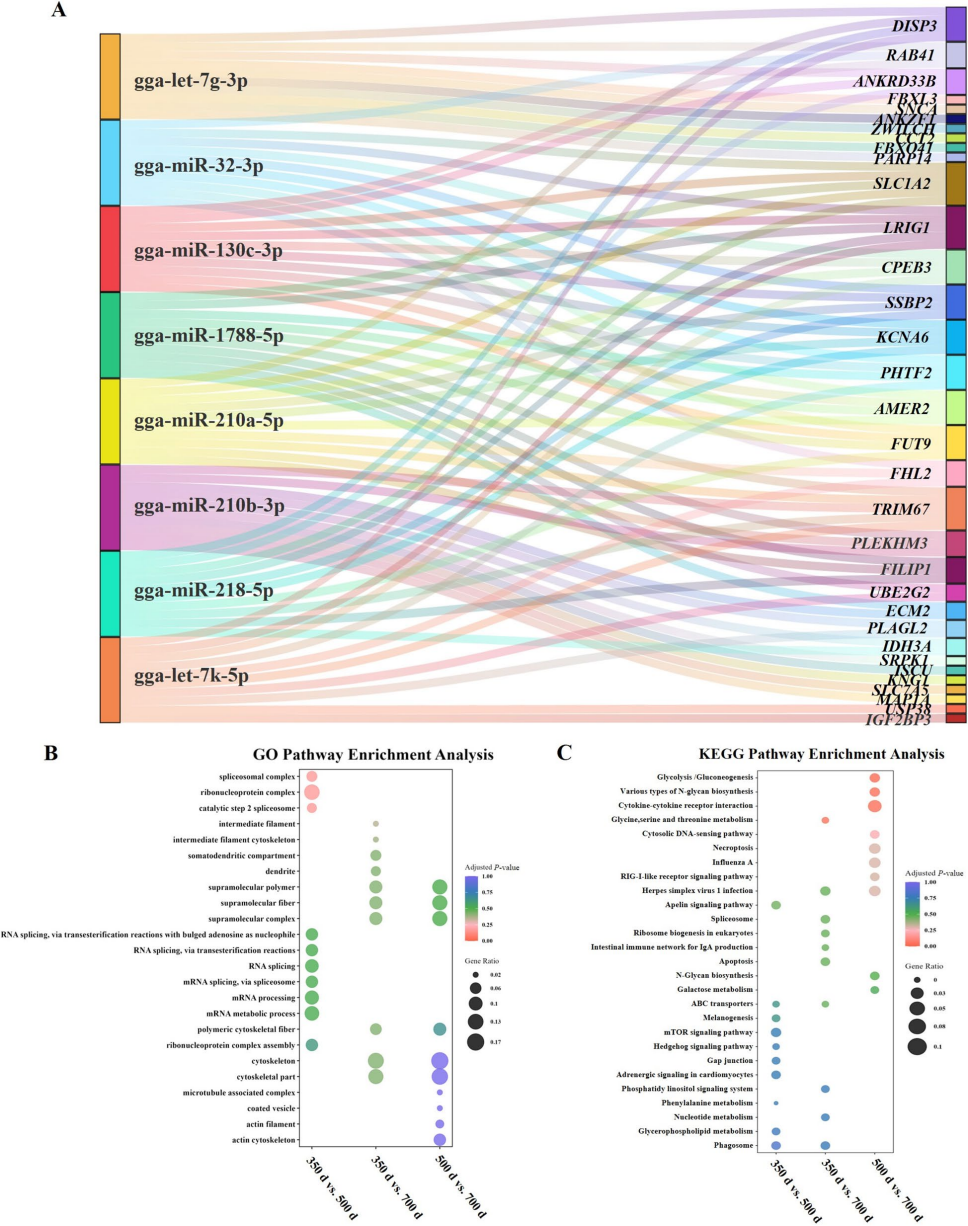
Functional analysis of target genes of DEMs. **A** Sankey diagram of DEMs and their target genes. **B** GO enrichment analysis of predicted target genes of DEMs. **C** KEGG pathway analysis of predicted target genes of DEMs

To explore the post-transcriptional regulation associated with uterine aging, a Sankey diagram was generated to visualize the interactions between DEMs and their predicted mRNA targets (Fig. 5A). The diagram reveals a complex post-transcriptional regulatory network. Notably, several miRNAs, such as gga-let-7k-5p and gga-miR-210a-5p, were linked to numerous genes involved in cellular senescence, metabolism, and signal transduction, including *FHL2*, *CPEB3*, and *IGF2BP3*. Moreover, the convergence of different miRNAs on shared targets (e.g., *SLC1A2*, *LRIG1*, *TRIM67*) suggests coordinated regulation of aging-related pathways in uterine tissues. GO enrichment analysis of miRNA target genes revealed significant Functional changes related to RNA splicing And processing between 350 And 500 d, including enrichment of terms such as spliceosomal complex, catalytic step 2 spliceosome, And mRNA splicing via spliceosome. In contrast, between 500 And 700 d, enrichment was observed in cellular components associated with cytoskeletal structures, including intermediate filament, intermediate filament cytoskeleton, polymeric cytoskeletal fiber, and microtubule-associated complex (Fig. 5B). KEGG pathway Analysis indicated that changes from 350 to 500 d were mainly associated with cell communication, metabolism, And transport, including gap junction, melanogenesis, ABC transporters, And the apelin signaling pathway. while changes from 500 to 700 d were linked to energy metabolism and immune responses, including the glycolysis/gluconeogenesis, cytosolic DNA-sensing pathway, various types of N-glycan biosynthesis, cytokine-cytokine receptor interaction (Fig. 5C).

### Integrated analysis of mRNA and miRNA sequencing data

**Fig. 6:**
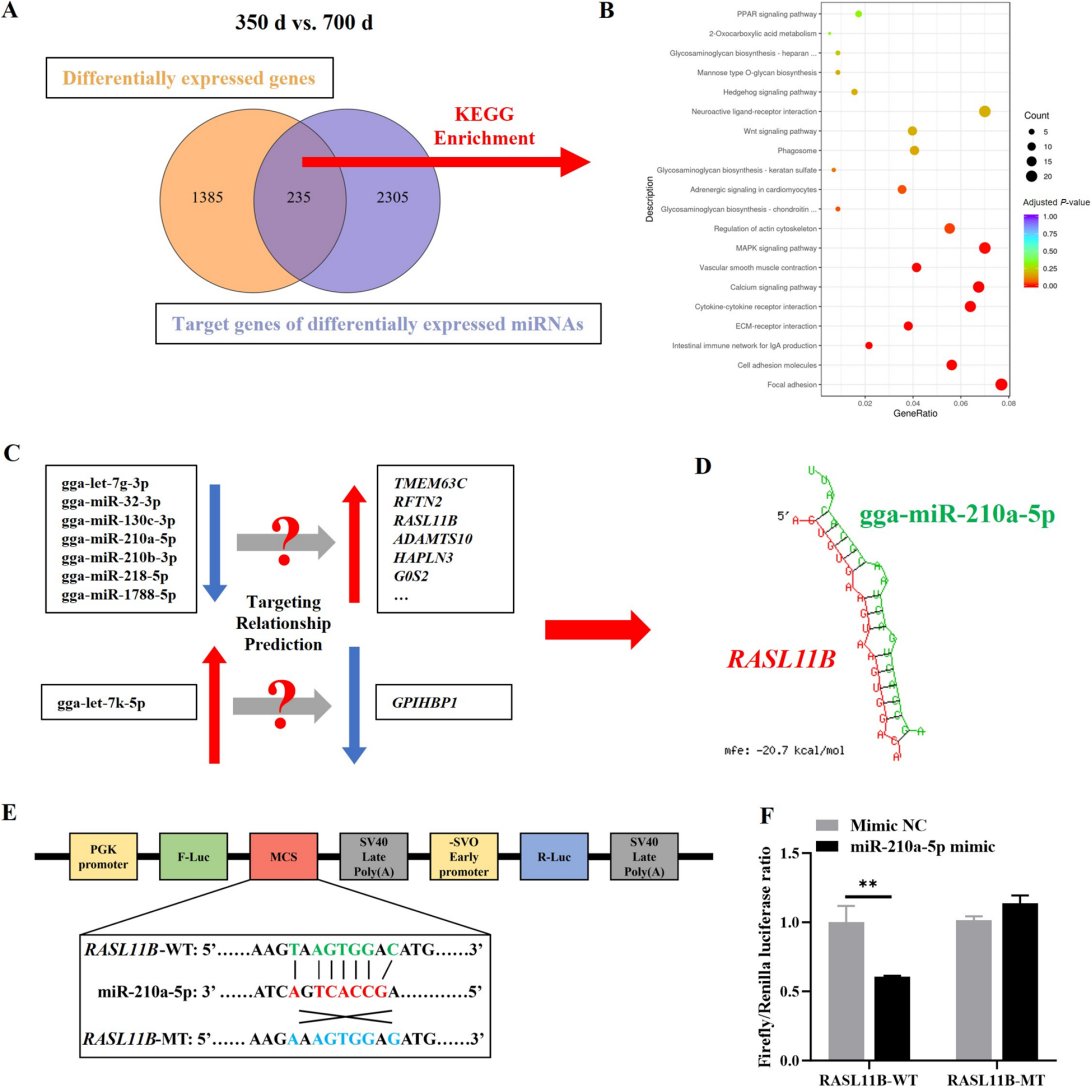
Integrated analysis of mRNA and miRNA sequencing reveals miR-210a-5p targets *RASL11B* during uterine aging. **A** Venn diagram showing the overlap of DEGs from mRNA-seq with predicted target genes of DEMs between 350 And 700 d. **B** KEGG pathway enrichment Analysis of the 235 overlapping genes. **C** Prediction of targeting relationships between inversely expressed DEMs and DEGs. **D** Molecular hybridization analysis of miR-210a-5p and *RASL11B* interaction. **E** WT and MT sequences of *RASL11B* binding sites with miR-210a-5p. **F** Dual-luciferase reporter assay analyzing the targeting relationship between miR-210a-5p and *RASL11B*

To Further investigate miRNA-mediated regulation of mRNA expression during uterine aging, we performed An integrated Analysis of mRNA-seq and miRNA-seq data. Venn diagram analysis revealed that 235 predicted target genes of DEMs between 350 And 700 d were also differentially expressed in the mRNA-seq data (Fig. 6A). KEGG pathway enrichment analysis of these genes highlighted significant involvement in cell adhesion, ECM-receptor interaction, MAPK signaling pathway, calcium signaling pathway and inflammatory pathways, suggesting miRNA regulation of these critical pathways during uterine aging (Fig. 6B). Target prediction analysis identified miR-210a-5p as a regulator of *RASL11B*, with miR-210a-5p expression decreasing and *RASL11B* expression increasing with age (Fig. 6C and D). Dual-luciferase reporter assays confirmed that miR-210a-5p directly targets the 3′ UTR of *RASL11B*, indicating its role in modulating uterine aging through *RASL11B* regulation (Fig. 6E and F).

### miR-210a-5p inhibits senescence in chicken UECs

**Fig. 7:**
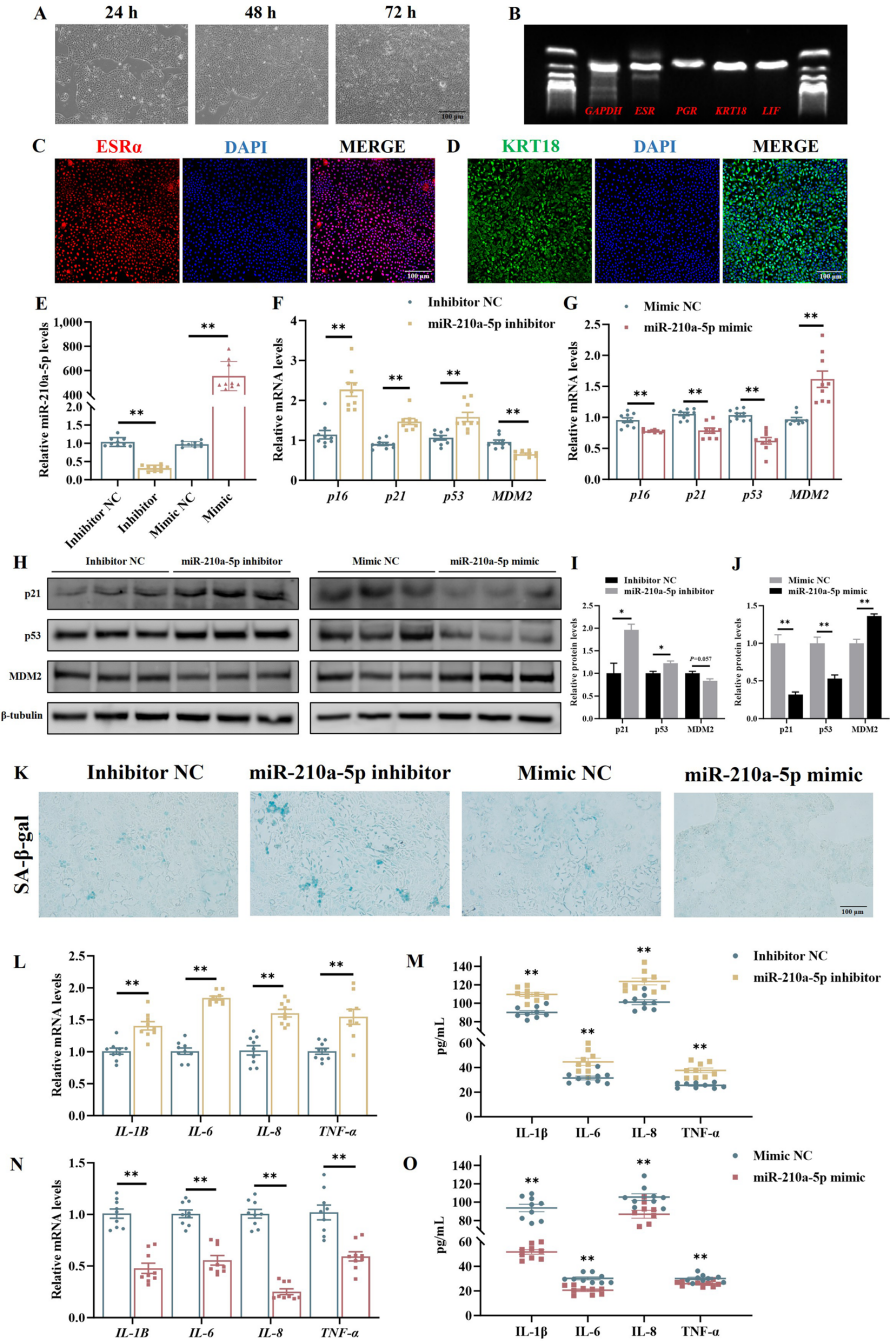
miR-210a-5p suppresses senescence and inflammation in UECs. **A** Morphology of UECs at 24, 48, And 72 h post-isolation. **B** PCR showing epithelial-specific gene expression. **C** and **D** Immunofluorescence staining confirming high expression of ESRα and KRT18 in cultured UECs. **E** Relative expression levels of miR-210a-5p detected by qPCR after transfection with miR-210a-5p inhibitor or mimic. **F** and **G** The mRNA expression levels of senescence-related genes (*p16*, *p21*, *p53*, and *MDM2*) measured by qPCR after transfection with miR-210a-5p inhibitor or mimic. **H**–**J** Protein expression levels of p21, p53, and MDM2 assessed by Western blotting; band intensities were quantified using ImageJ. **K** Cellular senescence detected by SA-β-gal staining. **L**–**O** Expression levels of pro-inflammatory cytokines (*IL-1B*, *IL-6*, *IL-8*, and *TNF-α*) analyzed by qPCR, and cytokine secretion quantified using ELISA after transfection with miR-210a-5p inhibitor or mimic. *P < 0.05, **P < 0.01

To investigate the role of miR-210a-5p in the senescence of chicken UECs, we successfully isolated and cultured UECs in vitro. As shown in Fig. 7A, the cells reached approximately 70% confluence at 24 h post-seeding, 90% at 48 h, And nearly 100% at 72 h. PCR Analysis confirmed the high expression of epithelial cell-specific genes, And IF staining revealed that more than 95% of the cells were positive for the uterine epithelial markers ESRα and KRT18, confirming both identity and high purity of the isolated cells (Fig. 7B–D).

Next, we transfected the cells with either miR-210a-5p mimic or inhibitor. qPCR analysis showed that the mimic significantly upregulated, while the inhibitor significantly downregulated miR-210a-5p expression in the cells (*P* < 0.01, Fig. 7E). Functional assays demonstrated that inhibition of miR-210a-5p led to a marked upregulation of senescence-associated genes *p16*, *p21*, and *p53* at the mRNA level, and increased protein levels of p21 and p53 (*P* < 0.01, Fig. 7H and I), accompanied by decreased expression of MDM2 at both transcript (*P* < 0.01, Fig. 7F) and protein levels (*P* = 0.057, Fig. 7H and I). In contrast, miR-210a-5p overexpression suppressed p16, p21, and p53, while upregulating MDM2 (*P* < 0.01, Fig. 7G–J). Furthermore, inhibition of miR-210a-5p significantly increased the proportion of SA-β-gal-positive area, a hallmark of cellular senescence, whereas overexpression reduced it (*P* < 0.01, Fig. 7K, Fig. S2). Importantly, knockdown of miR-210a-5p also elevated the expression of inflammatory cytokines *IL-1*, *IL-6*, *IL-8*, and *TNF-α* (*P* < 0.01, Fig. 7L), as well as their secretion (*P* < 0.01, Fig. 7M), while its overexpression exhibited the opposite effect (*P* < 0.01, Fig. 7N and O). These findings suggest that miR-210a-5p suppresses senescence and mitigates the SASP in chicken UECs.

### RASL11B promotes senescence in chicken UECs

**Fig. 8:**
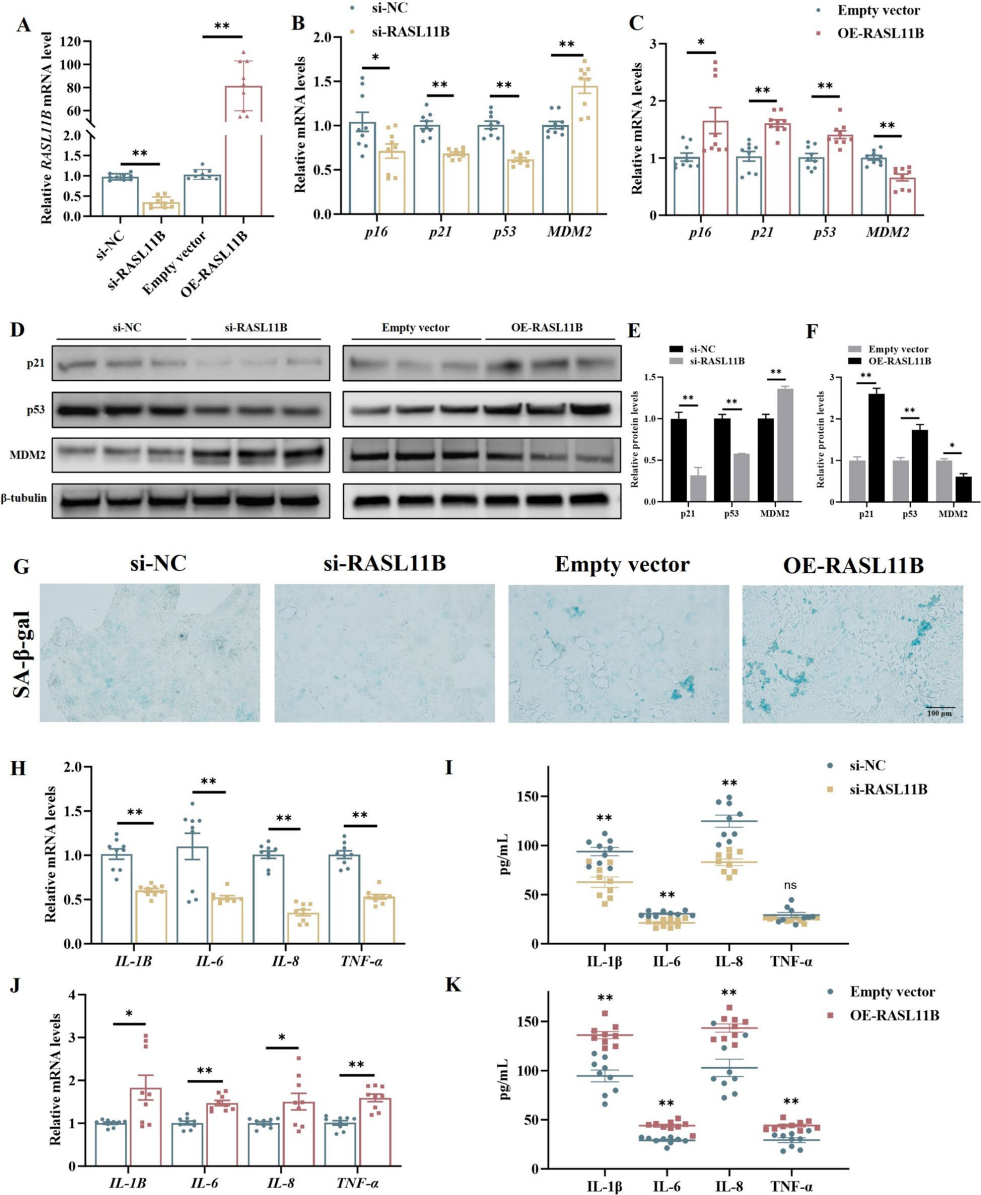
RASL11B facilitate senescence and inflammation in UECs. **A** Relative expression levels of *RASL11B* detected by qPCR after treatment with si-RASL11B or OE-RASL11B. **B** and **C** The mRNA expression levels of senescence-related genes (*p16*, *p21*, *p53*, and *MDM2*) measured by qPCR after treatment with si-RASL11B or OE-RASL11B. **D**–**F** Protein expression levels of p21, p53, and MDM2 assessed by Western blotting; band intensities were quantified using ImageJ. **G** Cellular senescence detected by SA-β-gal staining after treatment with si-RASL11B or OE-RASL11B. **H**–**K** Expression levels of pro-inflammatory cytokines (*IL-1B*, *IL-6*, *IL-8*, and *TNF-α*) analyzed by qPCR, and cytokine secretion quantified using ELISA after treatment with si-RASL11B or OE-RASL11B. *P < 0.05, **P < 0.01

To determine the function of RASL11B in cellular senescence, three siRNAs targeting *RASL11B* were designed and synthesized. Among them, siRNA-1 exhibited the highest knockdown efficiency and was used for subsequent experiments (*P* < 0.01, Fig. 8A, Fig. S3). qPCR results showed that silencing RASL11B significantly downregulated the mRNA levels of *p16*, *p21*, and *p53*, while upregulating *MDM2* expression (*P* < 0.01, Fig. 8B). Conversely, overexpression of RASL11B increased *p16*, *p21*, and *p53* expression and decreased *MDM2* levels (*P* < 0.01, Fig. 8C). And western blot results showed the same trend (*P* < 0.0, Fig. 8D–F). Moreover, knockdown of RASL11B also significantly reduced SA-β-gal-positive staining, whereas overexpression led to a notable increase (*P* < 0.05, Fig. 8G, Fig. S4). Additionally, RASL11B silencing diminished the expression of inflammatory cytokines (*P* < 0.01, Fig. 8H) and reduced inflammatory factor secretion (*P* < 0.01, Fig. 8I). In contrast, RASL11B overexpression enhanced both inflammation-related gene expression and secretion (*P* < 0.01, Fig. 8J and K). These results indicate that RASL11B acts as a pro-senescence factor in chicken UECs, with functions antagonistic to miR-210a-5p.

### miR-210a-5p regulates UECs senescence via the RASL11B/B-Raf/MAPK signaling cascade

**Fig. 9:**
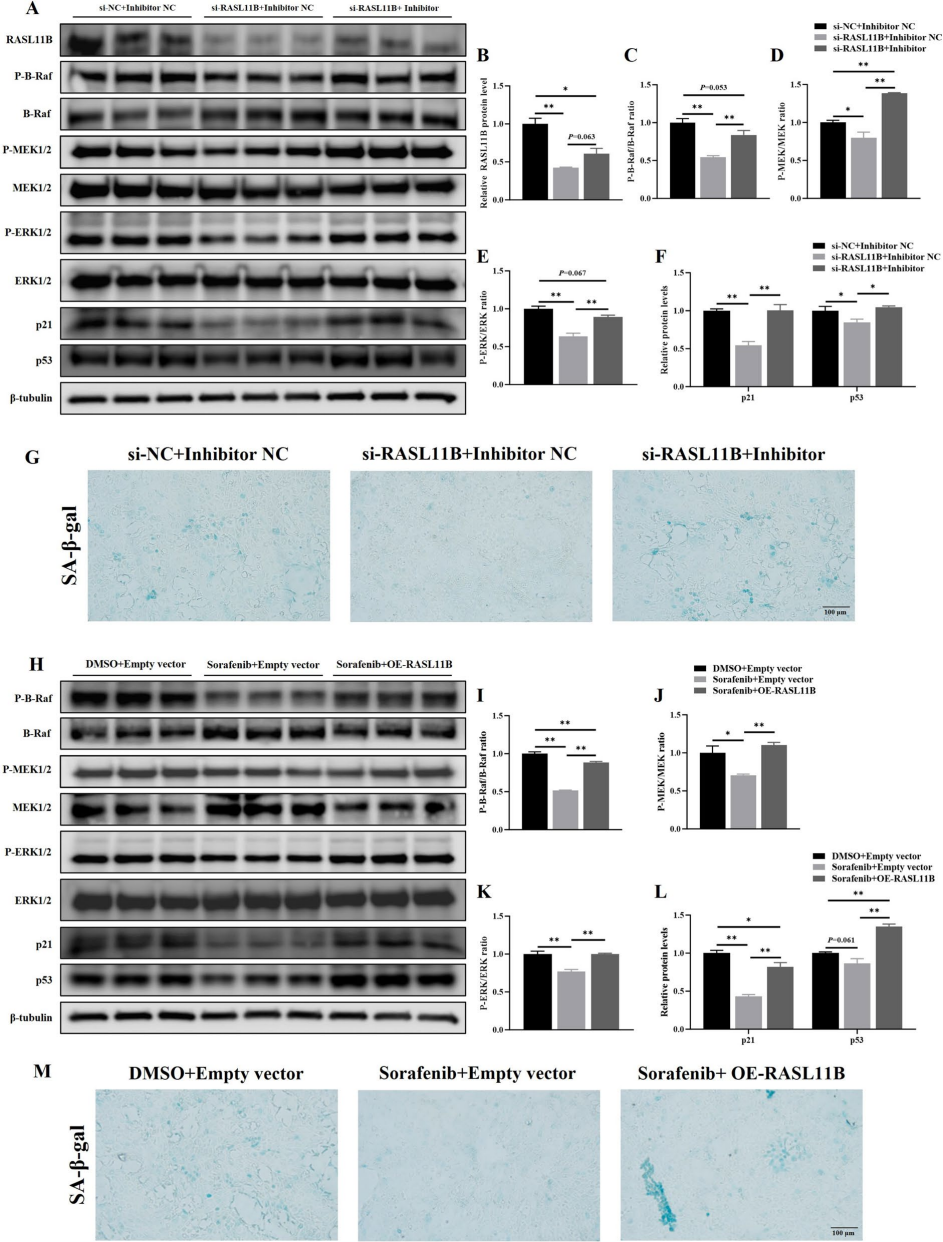
miR-210a-5p regulates MAPK signaling via the RASL11B/B-Raf/MEK/ERK cascade in UECs. **A**–**F** Western blot analysis of RASL11B, phosphorylated and total B-Raf, MEK, and ERK, p21, and p53 protein levels following co-transfection with miR-210a-5p inhibitor or NC and RASL11B siRNA or NC; band intensities were quantified using ImageJ. **G** SA-β-gal staining assay showed that inhibition of miR-210a-5p alleviated the reduction in cellular senescence induced by RASL11B knockdown. **H**–**L** Western blot analysis of phosphorylated and total B-Raf, MEK, and ERK, p21, and p53 protein levels following co-treatment with Sorafenib or DMSO and OE-RASL11B or empty vector; band intensities were quantified using ImageJ. **M** SA-β-gal staining assay showed that overexpression of RASL11B alleviated the reduction in cellular senescence induced by Sorafenib. *P < 0.05, **P < 0.01

RAS family proteins are well-established upstream regulators of the MAPK signaling pathway. To investigate whether the miR-210a-5p/RASL11B axis modulates MAPK signaling in chicken UECs, we conducted co-transfection experiments. Western blot analysis revealed that RASL11B knockdown decreased phosphorylation levels of B-Raf, subsequently reducing the phosphorylation of downstream MEK and ERK, which ultimately attenuated the senescence phenotype (*P* < 0.01, Fig. 9A–E). This was evidenced by reduced levels of senescence markers p21 and p53 (*P* < 0.01, Fig. 9A and F), and a lower percentage of SA-β-gal-positive area (*P* < 0.05, Fig. 9G, Fig. S5). Notably, inhibition of miR-210a-5p reversed these effects (*P* < 0.05, Fig. 9A–G, Fig. S4), suggesting that miR-210a-5p negatively regulates the RASL11B/B-Raf/MAPK axis. Furthermore, treatment with the Raf inhibitor Sorafenib significantly decreased MEK/ERK phosphorylation and downregulated p21 and p53 (*P* < 0.01, Fig. 9H–L), alongside reduced SA-β-gal staining (*P* < 0.05, Fig. 9M, Fig. S6). Overexpression of RASL11B partially reversed Sorafenib-induced suppression of MAPK activity and senescence attenuation (*P* < 0.05, Fig. 9H–M, Fig. S5). Collectively, these findings demonstrate that miR-210a-5p suppresses senescence in chicken UECs by directly targeting RASL11B, thereby inhibiting the activation of the B-Raf/MEK/ERK signaling cascade and mitigating cellular aging.

## Discussion

Aging of the reproductive system is a major factor contributing to decreased egg production and shell quality in poultry [22, 23]. In this study, we provide comprehensive evidence that miR-210a-5p serves as a key negative regulator of uterine epithelial cell senescence in chickens. Through multi-level functional assays and pathway analyses, we demonstrate that miR-210a-5p targets RASL11B and modulates the B-Raf/MAPK signaling axis to influence the SASP, ultimately affecting the uterine microenvironment. Our findings provide novel insights into the molecular basis of reproductive tract aging in poultry.

Eggshell quality, particularly shell strength and thickness, is a vital determinant of egg viability and commercial value [24, 25]. Our results show a significant decline in these traits in aged hens, concurrent with increased expression of cellular senescence markers (*p21*, *p53*) in the uterine epithelium. This correlation suggests that uterine epithelial senescence contributes directly to reduced shell formation capacity. Previous studies have indicated that the shell gland epithelium is responsible for secreting matrix proteins and regulating calcium deposition, both essential for proper shell mineralization [26, 27]. With aging, functional deterioration of epithelial cells may impair these processes, resulting in compromised shell structure. These observations highlight the importance of maintaining uterine epithelial integrity to sustain reproductive performance in aging hens.

The transcriptomic Analysis of the uterine segment across different ages in laying hens provides compelling evidence of progressive molecular changes associated with reproductive aging. Our mRNA-seq results show a dynamic shift in gene expression profiles from 350 to 500 d, indicating a clear transcriptional reprogramming that underlies the decline in uterine function during the aging process. Firstly, the global gene expression trends revealed a slight but consistent decrease in overall transcriptional activity with age, which aligns with previous findings in mammalian reproductive tissues where aging is often accompanied by a global transcriptional slowdown [28, 29]. The heatmap And PCA results clearly delineate age-related divergence in gene expression, particularly between 350 And 500 d, marking this interval as a critical window for the onset of uterine senescence. This separation in the PCA plot reflects the onset of distinct physiological and molecular states, suggesting that uterine aging is not a gradual linear process but rather accelerates during specific temporal windows [30]. To assess the reliability of our differential expression analysis, we evaluated both the statistical power and the FPR using the PROPER R package. The results indicated a low FPR (~ 0.0078) but limited statistical power (~ 0.04), likely due to the small number of replicates. In contrast, DESeq2 estimated a higher theoretical power (~ 0.8). Despite the low simulated power, a substantial number of differentially expressed genes were identified, suggesting strong underlying biological signals. Key aging-associated genes such as *p53* and *ATM* were significantly upregulated, indicating increased DNA damage response and cellular stress [31]. Concurrently, genes linked to mitochondrial function, cell cycle regulation, and homeostasis were downregulated, pointing to diminished metabolic capacity and proliferative potential [32]. The upregulation of SASP-related genes at 500 And 700 d further supports the presence of a senescent secretory phenotype, likely contributing to local inflammation and tissue degeneration [33]. These molecular events collectively compromise uterine Function And may underlie the decline in eggshell quality And laying performance during late reproductive stages. Interestingly, uniform expression patterns of senescence- and SASP-associated genes were observed in the 350 d chickens, whereas greater inter-individual variability was noted in the 500 And 700 d groups. This divergence may reflect increased biological heterogeneity with advanced age, a phenomenon commonly reported in aging tissues [34]. As senescence progresses, cells accumulate damage at variable rates due to differences in hormonal exposure, oxidative stress, and local tissue microenvironments, which can result in asynchronous activation of senescence pathways across individuals [35]. The heterogeneity in gene expression at later stages may thus represent the stochastic nature of aging and the differential onset or progression of cellular senescence among animals. The GO and KEGG enrichment analyses revealed several aging-associated changes in key biological processes and pathways. Ribosome-related processes were significantly enriched, suggesting alterations in protein synthesis machinery, which is consistent with the decline in translational fidelity and proteostasis observed during aging [36, 37]. ECM organization and cell adhesion reflects age-related changes in tissue structure, elasticity, and cell-matrix interactions, contributing to reduced regenerative capacity and increased tissue stiffness [38]. The oxidative phosphorylation pathway was also enriched, indicating potential mitochondrial dysfunction and impaired energy metabolism—hallmarks of aging that contribute to oxidative stress and cellular damage [39]. Immune-related pathways, including cytokine-cytokine receptor interaction, point to immunosenescence and chronic low-grade inflammation often seen in the elderly [40]. Additionally, enrichment in MAPK and calcium signaling pathways suggests altered cell signaling dynamics, which may influence cell proliferation, stress response, and apoptosis during aging [41–43]. These findings collectively underscore the multifaceted molecular alterations associated with aging.

The miRNA-seq Analysis provided crucial insights into the post-transcriptional regulatory mechanisms underlying uterine aging in laying hens. The identification of markedly altered miRNA expression patterns, particularly between 350 And 500 d, aligns with mRNA-seq data that suggest this time window as a critical phase of transcriptomic reprogramming. The predominance of downregulated miRNAs during aging suggests a loss of post-transcriptional repression, thereby allowing increased expression of target genes involved in senescence and inflammation [44]. Notably, many of the miRNAs commonly altered across all three time points were associated with targets such as *IGF2BP3*, *TRIM67*, and *PLAGL2*, genes previously implicated in cellular aging, apoptosis, and epithelial maintenance [45–48]. The overlap of miRNA targets emphasizes a level of regulatory redundancy, which may serve to fine-tune gene expression during physiological aging or buffer against environmental fluctuations [49, 50]. GO and KEGG pathway analyses Further illuminate the functional implications of these miRNA shifts. The enrichment of cytoskeleton- And inflammation-related pathways indicates that structural remodeling And immune activation are key features of uterine aging. Importantly, the transition from 350 to 500 d appears to be governed primarily by changes in metabolism And signal transduction, while the 500–700 d interval is characterized by pathways associated with immune responses and viral mimicry. This shift likely reflects a transition from an adaptive to a degenerative phase of aging. The concurrent activation of glycolytic and cytokine signaling pathways in this context may suggest a metabolic-inflammatory feedback loop—a hallmark of aging tissues.

The integrated Analysis of mRNA And miRNA transcriptomes provides compelling evidence for miRNA-mediated regulation of gene expression during uterine aging. The identification of 235 overlapping genes between differentially expressed mRNAs and miRNA targets underscores the extensive post-transcriptional control exerted by miRNAs in this process. KEGG pathway enrichment revealed these genes to be significantly involved in neural signaling, metabolic adaptation, and inflammation—hallmarks of age-related functional decline. These findings not only validate the physiological relevance of the observed transcriptomic changes but also suggest a coordinated regulatory network in which miRNAs orchestrate the transcriptional landscape of the aging uterus. Of particular significance is the identification of the miR-210a-5p/RASL11B axis. The inverse expression patterns of miR-210a-5p (downregulated) and RASL11B (upregulated) with advancing age, coupled with dual-luciferase assay confirmation of direct binding, strongly support a functional interaction. Given RASL11B's emerging role in senescence and stress response, its post-transcriptional repression by miR-210a-5p may represent a critical modulatory mechanism delaying the onset of uterine aging. This is further supported by the enrichment of MAPK and inflammatory pathways among the target genes, suggesting that miR-210a-5p may act as a molecular brake on age-associated pro-senescent signaling cascades via RASL11B regulation.

Our study revealed that miR-210a-5p plays a protective role against cellular senescence in chicken UECs. Overexpression of miR-210a-5p significantly downregulated the expression of key senescence markers such as p16, p21, and p53, and upregulated MDM2, an important negative regulator of p53 [51]. Conversely, inhibition of miR-210a-5p enhanced the expression of senescence-associated genes and increased the number of SA-β-gal-positive cells, suggesting accelerated cellular aging. These results indicate that miR-210a-5p negatively regulates the senescence phenotype, potentially through modulation of the p53-MDM2 axis. Furthermore, we observed that knockdown of miR-210a-5p led to a marked increase in the expression and secretion of inflammatory cytokines including IL-1β, IL-6, IL-8, and TNF-α, whereas its overexpression had the opposite effect, further confirming the anti-inflammatory potential of miR-210a-5p. These findings are consistent with previous studies in mammalian systems. For example, miR-210 has been shown to alleviate inflammatory damage in pig hip artery endothelial cells by targeting the NF-κB signaling pathway [52]. Similarly, Bei et al. reported that miR-210 protected the mouse heart from ischemia/reperfusion injury through inhibition of NF-κB activation [53]. The convergence of our results with these studies suggests a conserved function of miR-210 in regulating inflammation and cellular senescence across species. Given the known interplay between inflammation and aging, it is plausible that miR-210a-5p delays uterine aging, at least in part, by dampening pro-inflammatory signaling cascades such as NF-κB. These data collectively highlight the importance of miR-210a-5p as a key regulatory node in the maintenance of uterine epithelial cell homeostasis during aging.

Knockdown of RASL11B led to downregulation of p16, p21, and p53, coupled with enhanced expression of MDM2, while RASL11B overexpression produced the opposite effects. These data indicate that RASL11B positively regulates the p53 pathway, thereby promoting the onset and maintenance of the senescence program. Moreover, RASL11B elevated β-gal positivity and proinflammatory cytokine expression, reinforcing its role in driving both cellular aging and the SASP. The dual impact of RASL11B on both senescence and inflammation positions it as a key molecular node in uterine aging. Importantly, its expression was shown to be negatively regulated by miR-210a-5p, highlighting a regulatory axis with functional significance.

The functional role of RASL11B in cellular physiology remains incompletely defined. However, RASL11B is upregulated during TGF-β1-induced differentiation and in vascular inflammation models, pointing to its potential involvement in immune modulation and tissue remodeling [54]. These predicted functions support our findings, in which RASL11B positively regulates the p53 pathway, thereby promoting the onset and maintenance of the senescence program. Moreover, RASL11B elevated β-gal positivity and proinflammatory cytokine expression, reinforcing its role in driving both cellular aging and the SASP. The dual impact of RASL11B on both senescence and inflammation positions it as a key molecular node in uterine aging. Importantly, its expression was shown to be negatively regulated by miR-210a-5p, highlighting a regulatory axis with functional significance.

RASL11B, a member of the Ras-like family of small GTPases, was found to positively regulate the MAPK pathway [55], a well-known driver of cellular senescence in various contexts [56–59]. Mechanistically, RAS family GTPases—serve as molecular switches that, upon activation by upstream signals like receptor tyrosine kinases, exchange GDP for GTP and undergo conformational changes. These active RAS proteins then recruit and activate Raf kinases (e.g., B-Raf) by direct binding to their Ras-binding domain, initiating the MAPK cascade. B-Raf phosphorylates MEK1/2, which in turn activates ERK1/2, culminating in the regulation of downstream transcription factors that govern cell cycle progression, senescence, and inflammatory gene expression [60–62]. Consistent with this canonical mechanism, our results showed that knockdown of RASL11B inhibited the phosphorylation of B-Raf and downstream MAPK components MEK and ERK, leading to reduced expression of senescence markers such as p21 and p53, and decreased β-gal activity. The reversal of this anti-senescent effect upon miR-210a-5p inhibition further confirms the functional relevance of the miR-210a-5p/RASL11B axis. Notably, pharmacological inhibition of Raf using Sorafenib produced a similar anti-senescence outcome, which was partially reversed by RASL11B overexpression, suggesting that RASL11B mediates its pro-senescence effects primarily through the MAPK signaling pathway. These findings align with previous reports implicating MAPK signaling in epithelial aging and extend our understanding by identifying a specific miRNA upstream regulator that modulates this pathway [63–65]. Collectively, this study underscores the critical role of the miR-210a-5p/RASL11B/MAPK signaling module in controlling uterine epithelial homeostasis and offers a potential molecular target for ameliorating reproductive aging in poultry.

## Conclusion

**Fig. 10:**
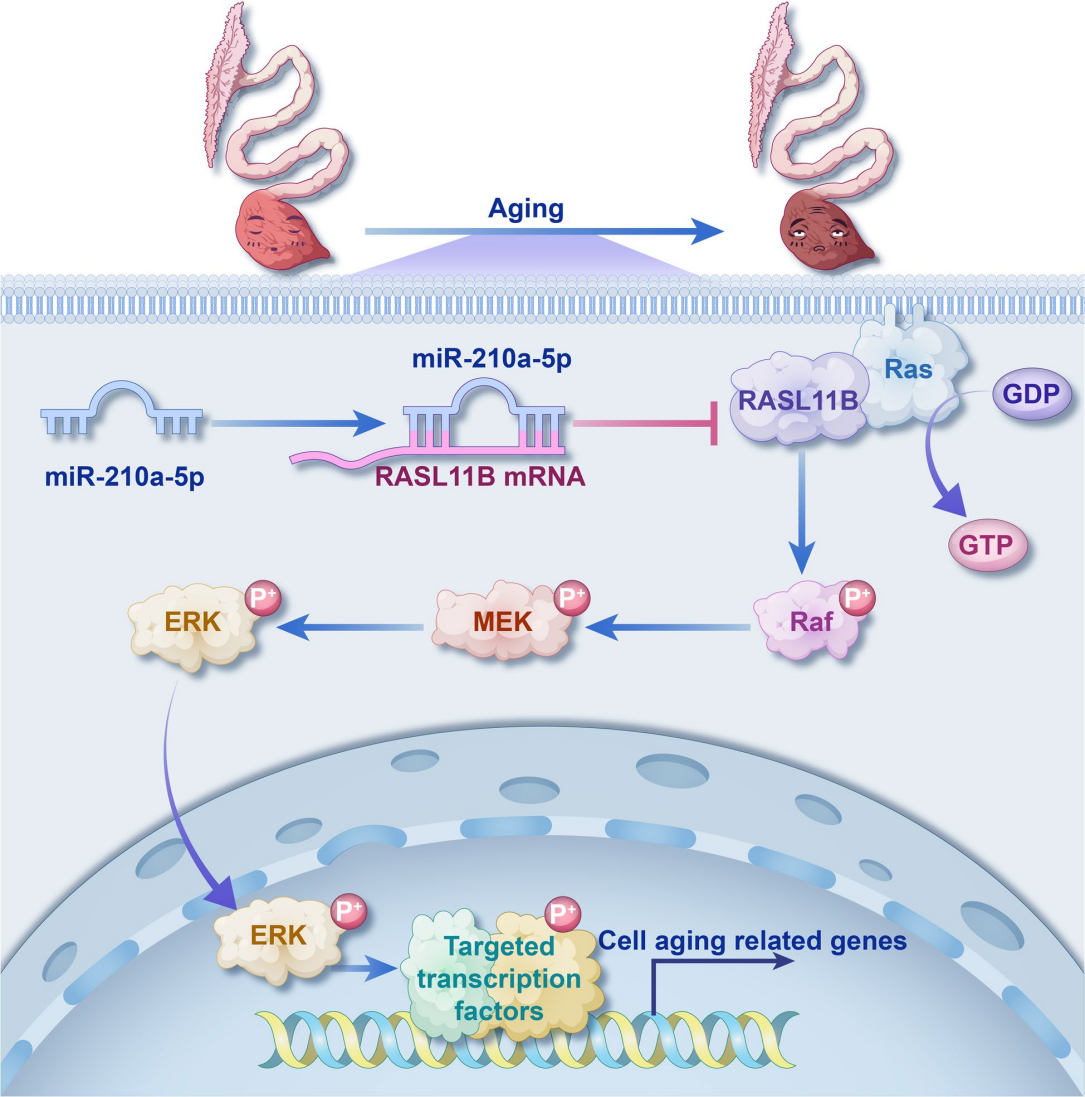
Schematic model illustrating the role of the miR-210a-5p/RASL11B/MAPK signaling axis in regulating UEC senescence. miR-210a-5p directly targets and suppresses the expression of RASL11B, a Ras-like GTPase that positively regulates the MAPK signaling pathway. Downregulation of RASL11B leads to decreased phosphorylation of B-Raf, MEK, and ERK, thereby attenuating MAPK pathway activation. This results in reduced expression of senescence markers (p21, p53), decreased SA-β-gal activity, and suppression of SASP-related inflammatory cytokines

This study identifies the miR-210a-5p/RASL11B/MAPK signaling axis as a key regulator of uterine epithelial senescence in laying hens. miR-210a-5p inhibits aging by targeting RASL11B, thereby suppressing MAPK pathway activation and downstream senescence responses (Fig. 10). These findings provide novel insights into the molecular mechanisms of reproductive aging and suggest potential targets for improving uterine function and extending laying performance in poultry.

## Supplementary Information

Additional file 1: Table S1 The antibodies used in this study. Table S2 Primers for qPCR in this study. Table S3 RNA oligonucleotides in this study.

Additional file 2: Fig. S1 Statistical analysis of the relative average optical density in immunohistochemical staining. Fig. S2 The relative positive area of β-gal staining after transfection with miR-210a-5p inhibitor or mimic. Fig. S3 The siRNAs interference efficiency of RASL11B. Fig. S4 The relative positive area of β-gal staining after treatment with si-RASL11B or OE-RASL11B. Fig. S5 The relative positive area of β-gal staining following co-transfection with miR-210a-5p inhibitor or NC and RASL11B siRNA or NC. Fig. S6 The relative positive area of β-gal staining following co-treatment with Sorafenib or DMSO and OE-RASL11B or empty vector.

## Data Availability

The mRNA-seq and miRNA-seq datasets have been deposited in the Sequence Read Archive (SRA) under accession number PRJNA1279392.

## Abbreviations

ceRNA: Competing endogenous RNA
DEGs: Differentially expressed genes
DEMs: Differentially expressed miRNAs
ELISA: Enzyme linked immunosorbent assay
FPKM: Fragments per kilobase of transcript per million mapped reads
FPR: False positive rate
GO: Gene Ontology
H&E: Hematoxylin and eosin
IF: Immunofluorescence
IHC: Immunohistochemistry
IL: Interleukin
KEGG: Kyoto Encyclopedia of Genes and Genomes
MAPK: Mitogen-activated protein kinase
miRNAs: MicroRNAs
miRNA-seq: MiRNA sequencing
mRNA-seq: MRNA sequencing
NC: Negative control
PBS: Phosphate buffer saline
PCA: Principal component analysis
qPCR: Quantitative real-time PCR
SASP: Senescence-associated secretory phenotype
SEM: Standard error of the mean
siRNAs: Small interfering RNAs
TPM: Transcripts per million
UEC: Uterine epithelial cell
β-gal: β-Galactosidase
3′ UTRs: 3′ Untranslated regions
