# Supplementary material for: Integrated mRNA-seq and miRNA-seq analysis reveals miR-210a-5p regulates uterine aging in laying hens by targeting the RASL11B/Raf/MAPK pathway
Source: J Anim Sci Biotechnol. 2025 Sep 23;16:129. doi: 10.1186/s40104-025-01257-y (PMC12455797; doi:10.1186/s40104-025-01257-y)
Supplement: Supplementary file 1 — Additional file 1: Table S1 The antibodies used in this study. Table S2 Primers for qPCR in this study. Table S3 RNA oligonucleotides in this study. [file 40104_2025_1257_MOESM1_ESM.docx]

**Table S1.** The antibodies used in this study

| Protein name | Supplier | Catalog No. | Dilution |
| --- | --- | --- | --- |
| β-Tubulin | Zenbio | 200608 | 1:5000 |
| P53 | ABclonal | A0263 | 1:1000 for WB 1:200 for IHC |
| Lamin B | ABclonal | A11495 | 1:200 |
| KRT18 | ABclonal | A1022 | 1:200 |
| ESRα | ABclonal | WX948351 | 1:200 |
| P21 | ABclonal | A1483 | 1:1000 |
| MDM2 | ABclonal | A13327 | 1:1000 |
| RASL11B | Biodragon | BD-PB2625 | 1:1000 |
| B-Raf | ABclonal | A2988 | 1:1000 |
| P-B-Raf | ABclonal | AP0012 | 1:1000 |
| MEK1/2 | ABclonal | A4868 | 1:1000 |
| P-MEK1/2 | ABclonal | AP0209 | 1:1000 |
| ERK1/2 | ABclonal | A4782 | 1:1000 |
| P-ERK1/2 | ABclonal | AP0472 | 1:1000 |
| HRP-conjugated Goat Anti-Mouse IgG | Zenbio | 511103 | 1:5000 |
| HRP-conjugated Goat Anti-Rabbit IgG | Zenbio | 511203 | 1:5000 |
| TRITC Goat Anti-Rabbit IgG | Zenbio | 511202 | 1:500 |
| FITC Goat Anti-Rabbit IgG | Zenbio | 511201 | 1:500 |

**Table S2.** Primers for qPCR

| Gene | Primer sequence |
| --- | --- |
| GAPDH | F: CCAGAACATCATCCCAGCGTC  R: ACGGCAGGTCAGGTCAACAA |
| P16 | F: CTGTTCCCATGACCTCTCGG  R: TCTTCTTCCGCGCTTCCTTG |
| P21 | F: ACGCGGGCAGACCACCATCAAA  R: AGGGAACTACAGACTCGGCATT |
| P53 | F: CGGTGCTGAATAAGGTCTAT  R: TCTCGTCGTCGTGGTAAC |
| MDM2 | F: TCTCATGGTGTGTGGTGAGC  R: TCCTCCTGACTGCTGCAAAG |
| IL-1β | F: AGTGAGGCTCAACATTGC  R: GTCCAGGCGGTAGAAGAT |
| IL-6 | F: CCTCCTCGCCAATCTGAA  R: CCTCACGGTCTTCTCCATA |
| IL-8 | F: CCTAACCATGAACGGCAAGC  R: CGTCCTACCTTGCGACAGAG |
| TNF-α | F: GACAGCCTATGCCAACAA  R: TCAACGCTCCTGACTCAT |
| RASL11B | F: GCTCAGTCACCTGTACCACC  R: AGAGGACGTGGAAGGCATTG |
| miR-210a-5p | AGCCACTGACTAACGCACATTG |
| U6 | GGGCCATGCTAATCTTCTCTGTA |
| miR-R | CAGGTCCAGTTTTTTTTTTTTTT |

**Table S3.** RNA oligonucleotides in this study

| Gene | Primer sequences (5'→3') |
| --- | --- |
| si-RASL11B-1 | F: GCGAACAAAGCUGACCUCUTT |
|  | R: AGAGGUCAGCUUUGUUCGCTT |
| si-RASL11B-2 | F: GAGGUUUAAGCAAGCUCUGTT |
|  | R: CAGAGCUUGCUUAAACCUCTT |
| si-RASL11B-3 | F: AUGGCUCUAGGAUGUAACUTT |
|  | R: AGUUACAUCCUAGAGCCAUTT |
| siRNA NC | F: UUCUCCGAACGUGUCACGUTT |
|  | R: ACGUGACACGUUCGGAGAATT |
| miR-210a-5p mimics | AGCCACUGACUAACGCACAUUG |
| mimics NC | UUGUACUACACAAAAGUACUG |
| gga-miR-210a-5p inhibitor | CAAUGUGCGUUAGUCAGUGGCU |
| inhibitor NC | CAGUACUUUUGUGUAGUACAA |
