# Supplementary material for: Integrated mRNA-seq and miRNA-seq analysis reveals miR-210a-5p regulates uterine aging in laying hens by targeting the RASL11B/Raf/MAPK pathway
Source: J Anim Sci Biotechnol. 2025 Sep 23;16:129. doi: 10.1186/s40104-025-01257-y (PMC12455797; doi:10.1186/s40104-025-01257-y)
Supplement: Supplementary file 2 — Additional file 2: Fig. S1 Statistical analysis of the relative average optical density in immunohistochemical staining. Fig. S2 The relative positive area of β-gal staining after transfection with miR-210a-5p inhibitor or mimic. Fig. S3 The siRNAs interference efficiency of RASL11B. Fig. S4 The relative positive area of β-gal staining after treatment with si-RASL11B or OE-RASL11B. Fig. S5 The relative positive area of β-gal staining following co-transfection with miR-210a-5p inhibitor or NC and RASL11B siRNA or NC. Fig. S6 The relative positive area of β-gal staining following co-treatment with Sorafenib or DMSO and OE-RASL11B or empty vector. [file 40104_2025_1257_MOESM2_ESM.docx]

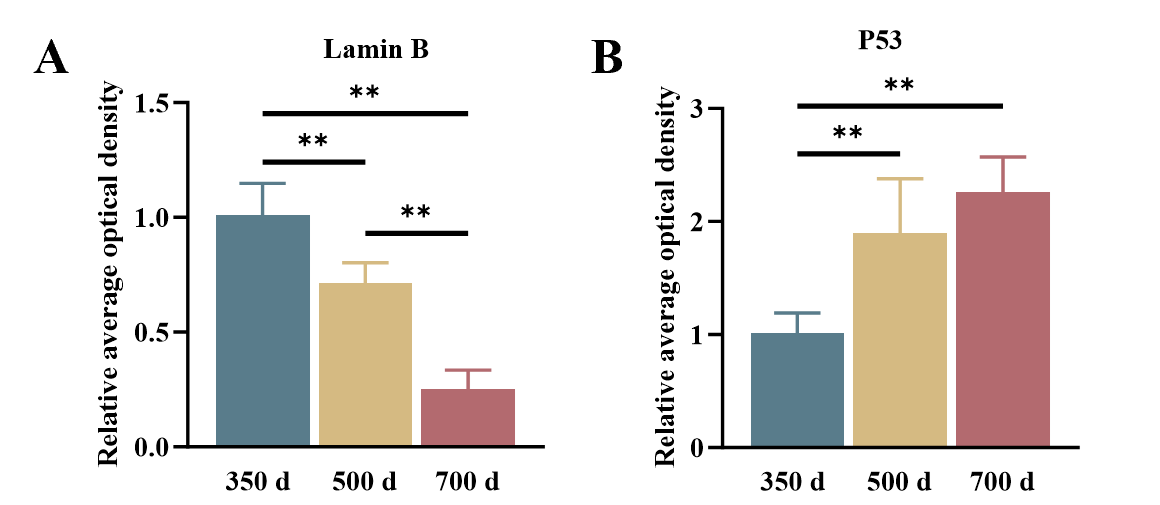


**Fig. S1** Statistical analysis of the relative average optical density in immunohistochemical staining. **A** Lamin B protein. **B** P53 protein.


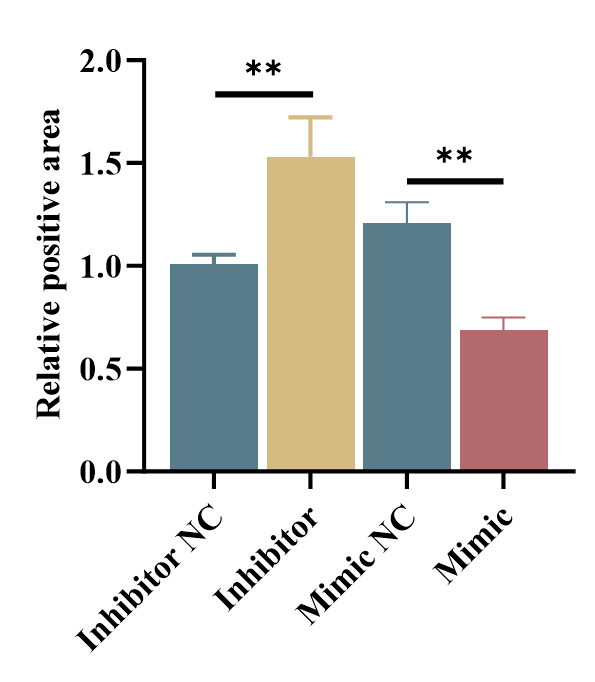


**Fig. S2** The relative positive area of β-gal staining after transfection with miR-210a-5p inhibitor or mimic.


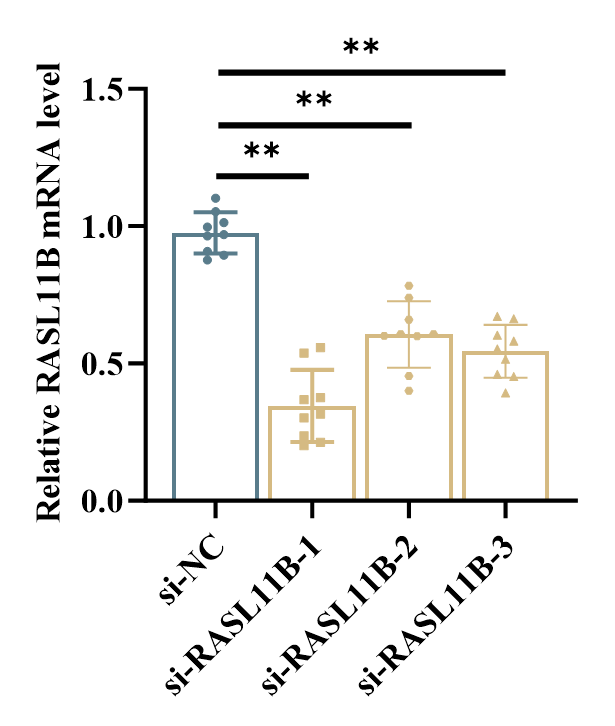


**Fig. S3** The siRNAs interference efficiency of RASL11B.


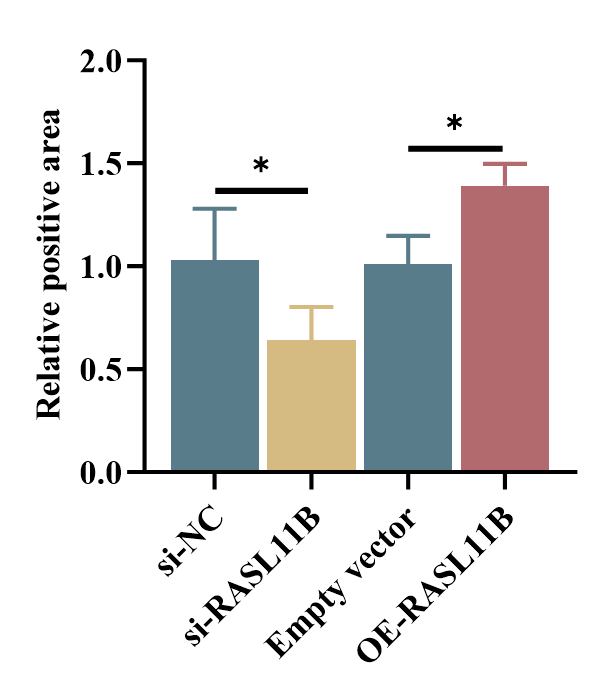


**Fig. S4** The relative positive area of β-gal staining after after treatment with si-RASL11B or OE-RASL11B.


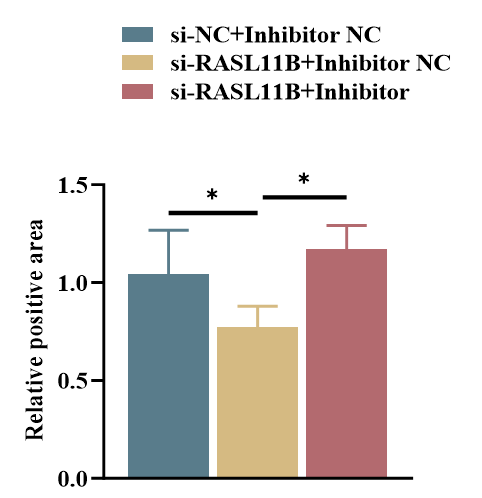


**Fig. S5** The relative positive area of β-gal staining following co-transfection with miR-210a-5p inhibitor or NC and RASL11B siRNA or NC.


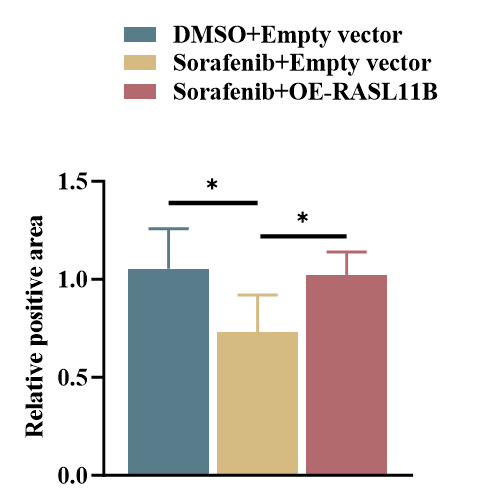


**Fig. S6** The relative positive area of β-gal staining following co-treatment with Sorafenib or DMSO and OE-RASL11B or empty vector.
